# A Quantitative Study of Gully Erosion Based on Object-Oriented Analysis Techniques: A Case Study in Beiyanzikou Catchment of Qixia, Shandong, China

**DOI:** 10.1155/2014/417325

**Published:** 2014-01-29

**Authors:** Tao Wang, Fuhong He, Anding Zhang, Lijuan Gu, Yangmao Wen, Weiguo Jiang, Hongbo Shao

**Affiliations:** ^1^College of Geography and Planning, Ludong University, Yantai 264025, China; ^2^Shandong Provincial Key Laboratory of Soil Conservation and Environmental Protection, Linyi 276000, China; ^3^Institute of Systems Science and Mathematics, NAAU, Yantai 264001, China; ^4^School of Geodesy and Geomatics, Wuhan University, Wuhan 430072, China; ^5^State Key Laboratory of Earth Surface Processes and Resource Ecology, Beijing Normal University, Beijing 100875, China; ^6^Key Laboratory of Coastal Biology & Bioresources Utilization, Yantai Institute of Coastal Zone Research, Chinese Academy of Sciences (CAS), Yantai 264003, China; ^7^Institute of Life Sciences, Qingdao University of Sciences & Technology (QUST), Qingdao 266042, China

## Abstract

This paper took a subregion in a small watershed gully system at Beiyanzikou catchment of Qixia, China, as a study and, using object-orientated image analysis (OBIA), extracted shoulder line of gullies from high spatial resolution digital orthophoto map (DOM) aerial photographs. Next, it proposed an accuracy assessment method based on the adjacent distance between the boundary classified by remote sensing and points measured by RTK-GPS along the shoulder line of gullies. Finally, the original surface was fitted using linear regression in accordance with the elevation of two extracted edges of experimental gullies, named Gully 1 and Gully 2, and the erosion volume was calculated. The results indicate that OBIA can effectively extract information of gullies; average range difference between points field measured along the edge of gullies and classified boundary is 0.3166 m, with variance of 0.2116 m. The erosion area and volume of two gullies are 2141.6250 m^2^, 5074.1790 m^3^ and 1316.1250 m^2^, 1591.5784 m^3^, respectively. The results of the study provide a new method for the quantitative study of small gully erosion.

## 1. Introduction

Soil erosion is a significant environmental issue of common concern in the world today, serious water and soil loss resulting from what has become one of the main factors restraining local economic development [1–3]. As a common type of soil erosion, though not of as wide range as surface erosion, however, because of its large amount and fast speed, gully erosion cuts complete slope into an area of scattered small sloping, decreasing farmland area and causing very serious harm to agricultural production, which has become a major source of river sediment [4, 5]. A series of studies showed that the gully-based valley sediment yield accounted for 10% to 94% of the total watershed sediment yield [2].

Since Rubey's study on gully erosion caused by ground subsidence in 1928, a lot of studies on gully erosion have been conducted, but gully erosion process is still not clear [6]. The establishment of a reasonable and effective gully erosion monitoring system is the premise of further systematic studies on gully. At present, scholars all over the world mainly utilize the ground manual measuring method [7–10], field marks [11, 12], aerial photos and digital elevation model [12]; RTK-GPS measurement [4, 5], and so forth to have quantitative study on gully erosion evolution. Ground manual measurement makes use of a tape measure, microtopographic profilers, or ruler to directly assess the cross-sectional areas along the channels. This method is simple, direct, and at low cost which is suitable for short-term measurement, but it is time consuming and not suitable for long-term and large-scale measurement with disadvantages, such as that calculation accuracy depends on the complexity of the gully's form and the length of the measuring intervals. For field marks monitoring, concrete or iron marks are planted in corners around the gully head. This method requires planting iron or concrete marks, which may exacerbate the instability of the gully edge. In addition, it is difficult to measure the changes of shape and volume resulted from internal collapse and headward erosion of the gully and so forth. Thus, the calculation error of erosion amount is quite difficult to control. For digital elevation model (DEM) method, gully erosion amount is estimated by using multiperiod aerial photos and DEM data (generated using photogrammetric method or interpolation of contours maps) [12–14]. This method is fast and simple and easy to conduct periodic monitoring, but due to uncertainties of remote sensing data, there are limitations in building DEM data accuracy and its resolution (temporal resolution and spatial resolution), its calculation accuracy is limited. With the development of GPS technology, RTK-GPS technology with the characteristics of high-precision, low-cost, and simple operation has become a fast, efficient study means in gully erosion evolution, but there still exists the problem of heavy field work load [4, 5]. Geographical phenomenon has obvious characteristics of scale effect, while the remote sensing technology with the advantages of multispatial scale, multirepeated observation period, and being suitable for dynamic monitoring may facilitate the study on rule sets of gully development in different spatial and temporal scales, which is very suitable for large area gully mapping [15].

Study on interpretation of soil erosion using remote sensing images can be traced back to 1940 [16]. In the following few decades after that, many scholars had carried out studies on gully erosion through aerial photographs and photographic measurement techniques [12, 17, 18]. But earlier aerial photographs were only limited to visible images with low spatial resolution and the resolution was lower for study method on gullies that stayed on the visual interpretation. Since the emergence of aerial photographic measurement techniques in the 1960s, remote sensing technology has gone through more than 60 years. Today, remote sensing technology has made remarkable achievements. Contemporary remote sensing technology shares the characteristics of multiplatform, multisensor, high spatial resolution, high temporal resolution, and high spectral resolution. With the springing up of high spatial resolution remote sensing data, as spatial scales are different, there are obvious shortcomings for pixel based remote sensing information extraction technology: (1) pixel-based algorithms rely only on spectral information, which cannot resolve the impact of “same object different spectrum” and “same spectrum but different object” on classification, (2) pixel-based algorithms do not introduce the direct participation of human knowledge and expertise [19], (3) classification results are severely affected by “salt and pepper effect” [20, 21], and (4) pixel-based algorithms ignore the spatial relationship between geographic objects and texture information which are important features for distinguishing the target. The root of these shortcomings is that pixel-based classification techniques ignore that the pixel value not only reflects its corresponding earth surface information but also contains signals from adjacent pixels corresponding to the surface [22]. In order to fully exploit the high spatial resolution remote sensing data, the classification techniques of object-based image segmentation have been developed. Since the classification techniques of “object-oriented” remote sensing image were developed in 2000, they have been applied in various fields [14, 23–28]. However, few examples of object-oriented information extraction technology used in gully study can be found. Eustace et al. [14] extracted gully information through air lidar data of Australian Fitzroy watershed from twenty LiDAR transects (5000 × 275 m). Based on iknos and geoeye-1 satellite data, Shruthi et al. [29] extracted gully information of Sehoul commune region in Morocco of Africa for study and pointed out the fast and objective advantage of gully mapping on the basis of object-based techniques. However, in complex terrain farming area, there has been no experiment for extraction of gully information based on object-oriented technique through high spatial resolution optical DOM aerial photos.

This paper, taking the Beiyanzikou catchment as a study area, selected two sub-gullies, named Gully 1 and Gully 2, which were in complete development with terraced farmland on ridgeland as the study gullies and extracted gully shoulder line to have a quantitative study on gully erosion on the basis of high spatial resolution optical DOM images. The study objectives include the following.

1. Through the analysis of texture, spectral, and geometrical features of surface subjects on DOM aerial photos of the study area, the image segmentation is expected to be made based on multiscale segmentation techniques, and the image objects are to be classified by object classification rule sets based mainly on spectral values, geometric characteristics, and spatial relationship between image and object so as to effectively extract the study gully.
2. The experimental gully boundary points are supposed to be randomly measured by RTK-GPS along gully shoulder line, and the gully accuracy extracted from high spatial resolution DOM aerial images is to be evaluated by adjacency method.
3. The soil erosion area is to be calculated by the gully extracted from aerial photos, and based on the differential theory assumptions, the original terrain surface of the gullies are to be recovered with linear regression fitting for sequencing elevation values points in the gully class edge line, so as to calculate the soil erosion volume of the experimental gullyies by using cut/fill tool of ArcGIS Desktop 10.0 software package.
4. A new set of technical route is to be provided for fast quantification of small gully soil erosion parameters in farming areas based on high spatial resolution optical DOM data.

## 2. Methods

### 2.1. The Study Area

**Figure 1:**
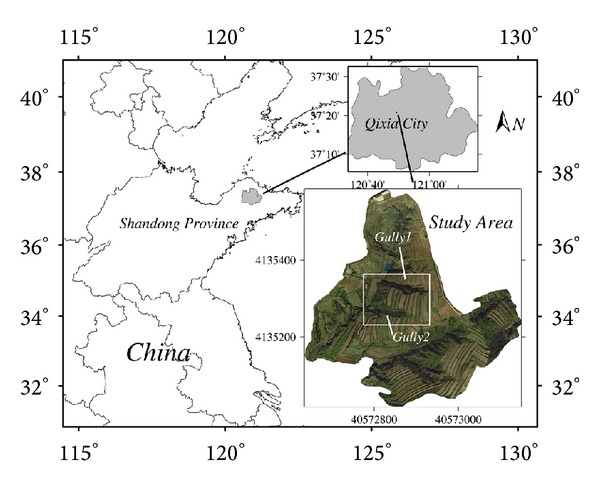
Location map of study gullies (Gully 1, Gully 2) at the Beiyanzikou catchmentin Qixia, eastern China.

Beiyanzikou catchment (120°49′E, 37°20′N) is a farming hills region in the northwest of Qixia City, Shandong Province of eastern China (Figure 1). The area is of hilly topography, with an altitude range of 130–180 m and an average of 175 m. There are many gullies developed within this area, with ravines cutting depth of 3–10 m, belonging to denudation and accumulation area. The gullies in the area, as aged gully, are in “U” shape, with artificial construction of terraces on both sides of the ditch, which destructed the original erosion features. Orchards and plantations are distributed on the ridgeland; grassland, shrub, and woodland are distributed on the gullied land. The main soil type in study area is brown soil covering 83.59% of the whole available acreage. The land between gullies in this area mainly includes orchards and farmland, and the land of gully-based valley mainly includes grassland, shrub, and woodland. The soil PH value and organic matter content of the main land-use type in this area have obvious distinctions: the soil PH values of farmland, orchards, fallow land, grassland, and woodland, respectively, are 6.20, 7.30, 6.67, 6.80, and 7.58; the mean values of soil organic matter content, respectively, are 2.752%, 3.181%, 3.933%, 4.101%, and 4.393%. The study area belongs to the warm temperate semi-humid monsoon climate, with annual average temperature of 11.3°C and annual rainfall of about 650 mm. The precipitation mainly appears in summer, and rainfall in July and August accounts for 57% of the annual precipitation, much of which is heavy rain and storm. The centralization and large annual variation of precipitation in study area are the main factors causing soil erosion.

### 2.2. Data

**Figure 2:**
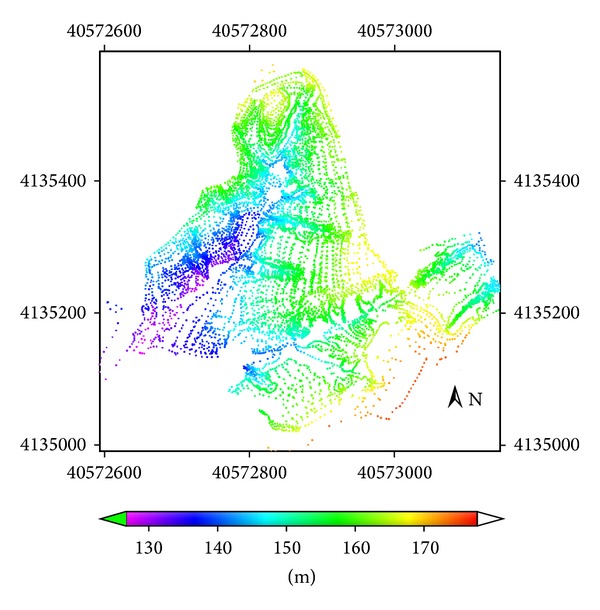
Distribution of RTK-GPS measured points in study area.

**Figure 3:**
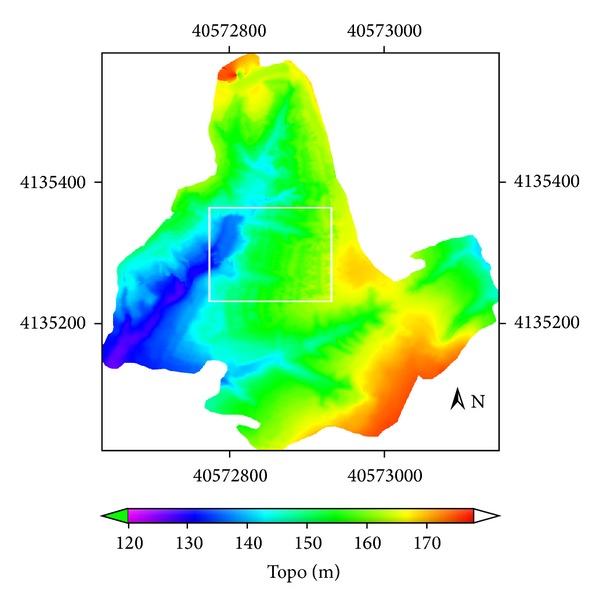
Digital elevation model of the study area.

**Figure 4:**
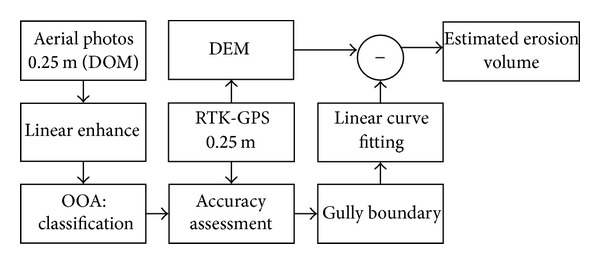
Flow chart showing the data and procedures followed for the study.

Data used in this study are referred to in Table 1. A topographic survey of the study area was made using a GPS system: Trimble 4700 RTK, with dynamic measurement and vertical precision of 10 mm ± 1 ppm and 20 mm ± 1 ppm, respectively. 10220 points were measured in the study area watershed (with area 0.1482328617 km^2^) from March 2008 to April 2008 (Figure 2). The digital elevation model (DEM) was created by Kriging interpolation (Figure 3) of the measured data. The pixel size of the DEM was 0.25 m. The image processing scheme is shown in Figure 4.

### 2.3. Image Preprocessing

The central wavelength and bandwidth setting of each band of the remote sensor determine its application. In addition, the amount of spectral information recorded by each type of bandwidth is not the same, and spectral redundancy and spatial redundancy are widespread in each band; therefore, the band should be chosen carefully. The band variance characterizes the degree of dispersion of grayscale which can reflect the image texture features, while correlation between bands describes the degree of spectral correlation between bands, which reflects the overlapping degree of information contained in bands. Therefore, appropriate band is supposed to be chosen to extract gully information on the basis of the band variance, correlation between bands and spectral characteristics of the ground object of the study area.

Due to the impact of environment, remote sensor performance, background, and other factors, the gray value distribution of remote sensing image is concentrated with louder noise, which results in the reduction of image distinguishability between different types of objects so that the overall image is degraded. In order to enhance spectral and texture features of the gullies and farming fields, the algorithm based on linear enhance method can be adopted for image enhancement processing.

### 2.4. Object-Oriented Classification of Gullies

#### 2.4.1. Multiscale Segmentation and Classification

The purpose of remote sensing image segmentation is to divide images into relatively homogeneous pixel groups which have certain semantics based on a certain scale. As geosciences have obvious scale effect characteristics, the segmented image object under a certain scale is only for a particular application [30]. Due to different scales of the gully, ridge-land, terrace ridge and other ground objects in the study area, multiresolution segmentation techniques are required for image multiresolution segmentation. The multiresolution segmentation algorithm locally minimizes the average heterogeneity of image objects for a given resolution of image objects [31]. Multiresolution segmentation parameters mainly include scale parameter and homogeneity criteria. The scale parameter is an abstract term that determines the maximum allowed heterogeneity for the resulting image objects. Homogeneity parameters consist of three criteria: color, smoothness, and compactness. Based on internal heterogeneity (homogeneity factor) minimum principle, similar adjacent pixels of spectral information are merged to form meaningful objects. In actual application, segmentation scale is determined by scale characteristics of the study object through repeated experiments.

In addition to the spectral information, object-oriented classification algorithm can also utilize the geometry of image object, object relative boundary, GLCM features, and so forth for classification.

Object relative boundary (relative border to neighbor objects) reflects adjacency relationship between objects. It is determined by the adjacency boundary length of an image object and the neighborhoods certain class of object divided by the total length of the boundary of the image object.

Texture refers to features that are partially irregular but macroscopically regular in images. Texture features are used to evaluate the texture of image objects and include features based on an analysis of subobjects helpful for evaluating highly textured data. GLCM (Gray Level Concurrence Matrix), extracting texture characteristics by conditional probability, makes statistics of grayscale relationship of a pair of pixels located at the same position and represents texture by certain grayscale of this pair. Representing texture by computing joint conditional probability density between each pixel grayscale within image object reflects the relationship of any two points of the image and statistics of texture characteristics. Two textural measures, that is, contrast and mean, were applied based on GLCM.

GLCM (Gray Level Concurrence Matrix) contrast is the opposite of homogeneity. It is a measure of the amount of local variation in the image [31]. The computational formula is

(1) $$\begin{array}{c} \text{GLC}{\text{M}}_{\text{Contrast}}=\sum_{i,j=0}^{N-1}P_{i,j}{\left(i-j\right)}^{2}. \end{array}$$

The feature values of GLCM contrast range from 0 to 65025.

GLCM (Gray Level Concurrence Matrix) mean measure reflects gray level mean within texture window, whose formula is

(2) $$\begin{array}{c} \text{GLC}{\text{M}}_{\text{MEAN}}=\frac{\sum_{i,j=0}^{N-1}P_{i,j}}{N^{2}}. \end{array}$$

The feature values of GLCM contrast range from 0 to 255. Consider

(3) $$\begin{array}{c} P_{i,j}=\frac{V_{i,j}}{\sum_{i,j=0}^{N-1}V_{i,j}}, \end{array}$$

where *i* is the row number; *j* is the column number; *V*_*i*,*j*_ is the value in the cell (*i*, *j*) of the matrix; *P*_*i*,*j*_ is the normalized value in the cell (*i*, *j*); *N* is the number of rows or columns [31].

When computing Gray Level Concurrence Matrix, it involves three major parameters: moving window size, step length, and direction. 0°, 45°, 90°, and 135° directions or mean are usually selected for moving direction. In this experiment, direction GLCM is adopted as the classification feature.

The geometry features of image object include aspect ratio, area, shape index of image object, and so forth, which are also the classification features applied in this study.

#### 2.4.2. Accuracy Assessment

In gully studies, the measuring accuracy of gully shoulder line directly affects the calculation of the volume of gully erosion. Therefore, accuracy assessment for gully edge of remote sensing classification is required. Error matrix, also called confusion matrix, is the most common way of expressing the accuracy of remotesensing image classifications. And the proportion of fitting and misclassification for various classifications between remote sensing classification and the ground inspection category can be checked out through confusion matrix.

There are two related limitations associated with accuracy assessments and error summaries calculated from the error matrix [32].

(1) The confusion matrix failed to offer spacial distribution information of classification error. (2) The overall accuracy measures derived from the error matrix may be inappropriate for subregions, where local error rates may be much smaller or larger than the global measures. Besides, when confusion matrix is taken as classification accuracy test, the reliability is greatly influenced by the selection of ground inspection category. As gully development rate varies greatly at different stages of gully development, 80% of the length, 50% of area, and 35% of volume of the gully system are formed only within 5% of the time in the life of gully system [33]. As aged gullies of the study area erosion surface widen slowly and no longer deepen, the amount of change in gully's area and volume in short period is usually not obvious; thus, accuracy assessment cannot be made by using confusion matrix for subtle change of the aged gully shoulder line. However, this study proposed a new approach of adjacency-based method to assess the extracted small gully shoulder line accuracy. In this method, firstly, a random field measurement of gully shoulder line was carried out to obtain data of the measured point at gully shoulder line. Then, a calculation of adjacent distance is to be conducted between measured point and classification boundary line extracted from remote sensing classification so as to obtain the distance value (deviation), and the mean value of deviation can be considered as the indicator of accuracy.

#### 2.4.3. Gully Volume

In gully erosion study, the amount of gully erosion is a very important parameter. As the experimental gullies (Gully 1, Gully 2) selected in this study are small gullies and there are flat farming fields of consistent terrain trend on both sides of the gullies; therefore, based on the mathematical differential theory, the elevation value of the points along the shoulder line of experimental gullies can be made use of with three-variable linear equation regression method to fit the original terrain surface before erosion of the experimental gullies. As a result, the erosion volume can be obtained with excavation square calculation on the basis of the fitted original surface and DEM data after erosion.

## 3. Results

### 3.1. Image Preprocessing

A linear method can be applied with a 2% clip on both ends of the DOM data to enhance image spectra and image texture. And statistical analysis is to be made for RGB triband of enhanced DOM data of linear 2% (Table 2). Red band has a high correlation between the green band and blue band while green and blue-ray correlation is weaker. In addition, because the visible blue light is most obviously affected by the atmosphere, it contributes little to the information extraction; visible green light can highlight the spectral differences of vegetation and arable land; the band variance of visible red light is the largest with texture information best presented and mainly reflects the soil spectral information. Thus, the red band spectrum is chosen as the main spectral band for follow-up image segmentation and classification.

**Figure 5:**
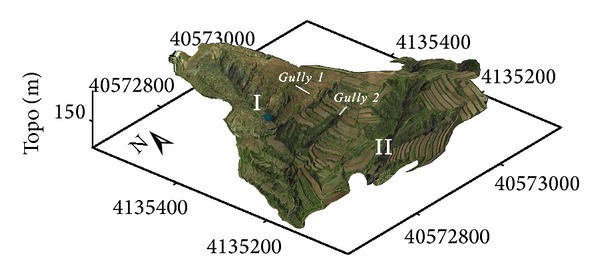
The DOM aerial imagery (red, green, and blue) overlaid on the DEM raster data.

The DOM aerial imagery (RGB: red, green, and blue) overlaid on the DEM raster data reconstructs the three-dimensional landscape of the study area (Figure 5). As Figure 5 shows, the experimental zone is composed of two main gullies—Gully 1 and Gully 2, and the ridgeland of the east side of Gully 1, consists of terraces on which orchards are distributed. Because the threshold in terms of flow hydraulics, rainfall, topography, pedology, and land use has been exceeded, the uniform farmland is cut with the formation of experimental gullies—Gully 1 and Gully 2.

### 3.2. Object-Oriented Classification of Gullies

A subset region (629 lines, 531 rows) of DOM image including experimental gullies (Gully 1, Gully 2) is used to conduct an object-oriented analysis. Based on the principle of different scales multiple segmentation, multiple classification, and multiclassification feature selection, assign class classification [31] is carried out by choosing spectrum (red), aspect ratio, relative boundary between the objects, area, and texture features (GLCM contrast and GLCM mean).

The gullies of the study area are covered with low vegetation, in which the north slope and southern slope of the sub-gully of Gully 2 basically share the same land using type, that is, sparse vegetation and regular farming fields. Thus the application of red spectral information and texture information may effectively distinguish farming fields. As low vegetation is grown in the agricultural field ridge, its spectral characteristics are similar to that of the gully, but the ridge has a regular rectangular shape feature and is distributed between the arable lands; therefore, ridge and gully can be distinguished by using geometry aspect ratio and relative boundary characteristics of the objects.

**Figure 6:**
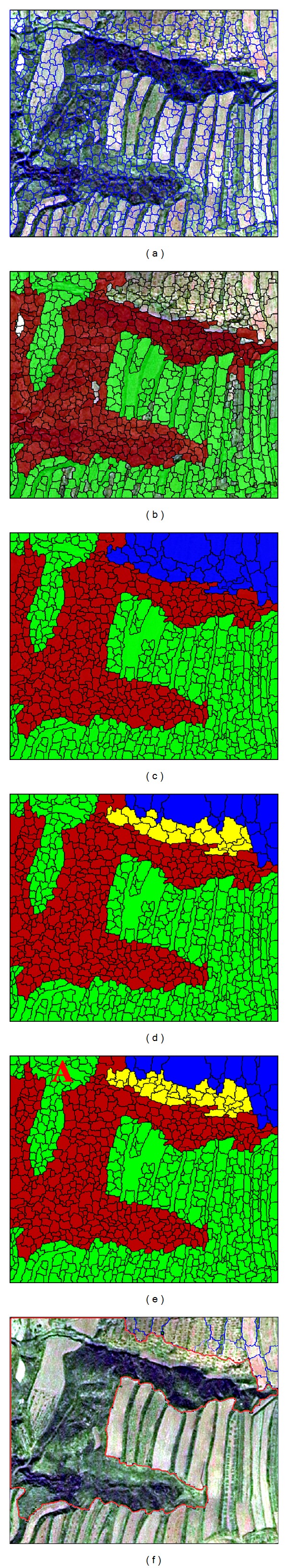
Steps of extracting gully erosion features using the OOA method.

For the first segmentation, one parameter of image layer weights is set as R: 1, G: 0.5, and B: 0. Other parameters are set as shape: 0.9, compactness: 0.8, and scale: 20 (Figure 6(a)). According to red band GLCM mean features (≤107), relative boundary (≥0.45), red band threshold (≤146) and other classes relative boundary features, the segmentation objects are classified into nongully class and gully class.  Figure 6(b) can basically distinguish Gully 2 from Gully 1, but northern slope of Gully 1 cannot be distinguished effectively (Figure 6(b) north-eastern corner). Part of Gully 1's farming information in southeast boundary is mistakenly classified as gully class, which can be categorized as farming class according to relative boundary features between image object and gully object (Figure 6(c)). In the experiment, relative boundary threshold is 0.2361. After gully class is merged, sporadic smallarea of the ridge patterns are still mistakenly classified as gully class, which can be weeded out according to the area attributes. The area parameter threshold selected in the experiment is 10000. In accordance with the gully class obtained (Figure 6(c) red patterns) after the first segmentation, the entire main gully has been completely overwritten. Due to segmentation scale, the northern edge line of Gully 1 is not clearly distinguished; therefore, a more appropriate scale analysis is needed in the follow-up second segmentation.

The second segmentation is made by taking image layer weights RGB: 1 : 1 : 0, shape: 0.9, compactness: 0.6, and scale: 30 as segmentation parameters (Figure 6(c) blue patterns). With segmentation scale of 30, north slope edge line of Gully 1 can be quite reasonably segmented. Then, classification can be made on the basis of the features of red band contrast GLCM (≥343) and relationship between image object and image boundary so that classification results of Figure 6(d) (yellow patterns) are finally obtained. As can be seen from Figure 6(d), as the second segmentation scale is larger, there is still a slight misclassification for north slope class at the edges of Gully 1 obtained after classification.

Next, a third segmentation-classification is made for classification results (yellow class in Figure 6(d) yellow patterns) of north slope of Gully 1 to refine the north slope edge. As sporadic fruit trees are planted in the north slope and the north of the gully is adjacent to fruit trees, compared with above segmentation, other parameters also need to be re-selected in addition to the segmentation scale parameter. Visible green ray can highlight spectral differences caused by the topography in fruit trees; density parameters can reflect the fruit density; the image textural features of fruit trees on north slope of Gully 1 are obviously different from those in the farming fields so that the shape parameter is also a very important segmenting factor. Thus, the third segmentation is made by taking image layer weights RGB: 0 : 1 : 0, shape: 0.8, compactness: 1, and scale: 30 as segmentation parameters (Figure 6(e) yellow patterns). Then, a further classification is made based on object relative boundary and red band spectral value features to obtain Figure 6(e). Besides, area *A* (farming class) lies at the bottom of Gully 1 in Figure 6(e), and it needs to be incorporated into gully class based on threshold of elevation value of image objects. Finally, as can be seen from the red line area in gully classification figure (Figure 6(f)), the main gullies in the north and south and the two sub-gullies, Gully 1 and Gully 2, in the study area are all accurately classified.

### 3.3. Accuracy Assessment

**Figure 7:**
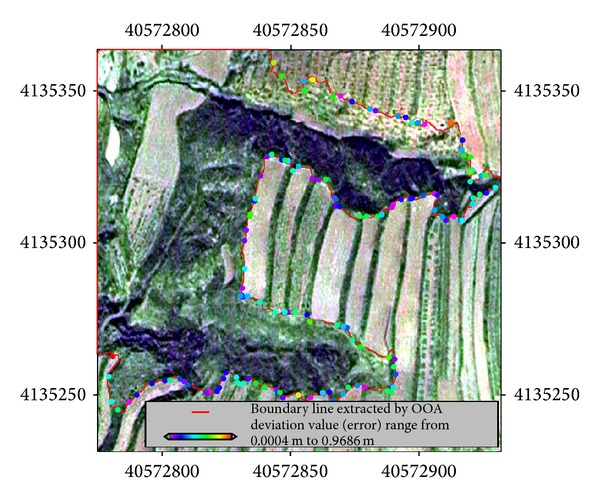
Comparison between gully boundary line extracted by OOA (red line) and RTK-GPS fielded measurement points coloured with the offset from gully boundary line extracted by OOA (red line).

An adjacent calculation and statistical analysis are successively conducted for randomly measured 144 points (points in Figure 7) of gully boundary in the field and gully boundary line (red line in Figure 7) extracted from remote sensing classification. The result shows that the minimum deviation value (error) is 0.0004 m, occupying 0.6944%, and the maximum deviation value is 0.9686 m, occupying 0.6944%. Submeter deviation accumulation accounts for 100%; the average error distance is 0.3166 m, the standard deviation is 0.2116 m, and reliability for the interval of 95% is 0.0349 m. As can be seen from above data, classification accuracy is quite high.

### 3.4. Gully Volume

Elevation value of each point on experimental gully boundaries is extracted by using ArcGIS Desktop 10.0 software package and SPSS17.0 software package is based to fit the experimental gully original surface:

(4) ZGully  1=0.113610741693193X −0.0632361956610761Y −4347856.65738527,ZGully  2=0.119824830773817X −0.00511127638780602Y −4840344.24784017.

The coefficient of determination (*R* ^2^) is 0.9461 and 0.8959, respectively. The residual mean square (RMS) is 0.5978 m and 0.6159 m, respectively. As can be seen from the fitting parameters, surface fitting accuracy is relatively high.

**Figure 8:**
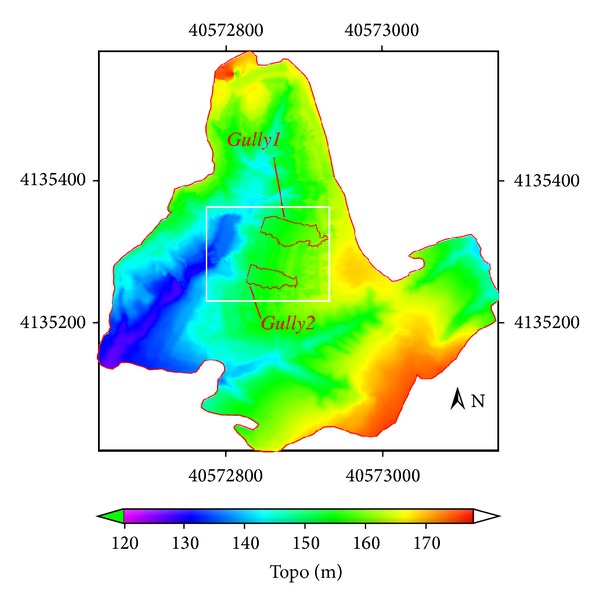
Fitting surface of study gullies (Gully 1, Gully 2) overlaid on the DEM raster data.

The cut/fill tool of ArcGIS Desktop 10.0 software package is used for fitting surface (Figure 8) data and DEM data (Figure 3) to obtain gully volume of experimental gullies (Gully 1, Gully 2), which is 5074.1790 m^3^ and 1591.5784 m^3^, respectively, and erosion area which is 2141.6250 m^2^ and 1316.1250 m^2^, respectively.

## 4. Discussion

The digital orthophoto map (DOM, digital orthophoto map) is a set of digital orthophoto generated from digital differential correction and inlay of air (or space) imagery, which is clipped according to a certain sheet range. The data is of map geometric accuracy and image features, which has a wide range of applications in the interpretation of geosciences. Using DOM data for effective gully interpretation involves a must for band choosing and necessary image enhancement in order to enhance the differences of different feature image spectroscopy, texture, and so on. In farming areas, data of visible red light, near-infrared bands, and normalized differential vegetation index (NDVI) should usually be considered to be chosen to enhance the spectral differences of soils between low shrubs and farming field in the gullies. The spectral information relied on in the image segmentation and classification of this paper is mainly red band, which has achieved better results. In addition, because many factors can lead to the loss of spectral information of the aerial photographs, including the imaging sensor performance, the band setting, imaging time, and imaging parameters, when conditions permit, corrections such as radiation, atmosphere, and terrain for the DOM aerial photographs are needed to restore the real spectral information.

Compared with pixel-based information extraction techniques, object-oriented analysis techniques can make gully classification under different segmentation scales based on spectral, geometry, and texture characteristics. Therefore, its application effect is better. In object-oriented image analysis, scale choosing is of great significance during the process of image segmentation, which has a direct and decisive influence on image size and accuracy of information extraction. Specific dimension is required in multidimensional segmentation of each image. The optimal value of segmentation dimension is that the boundary of this geographical object type can be made quite clear in the segmented polygon, and this geographical object can be shown with one object or several objects neither in a broken way nor with an obscure boundary. But in fact, spectrum information distortion of remote sensing image, distribution of geographical objects in earth's surface, and sensor difference all lead to the subjectivity to choose best thresholds (such as image segmentation scale factor and homogeneity criterion) and the attributes that most accurately classified gullies; for example, based on Light Detection and Ranging (LiDAR) and DEM (Digital Elevation Models generated by LiDAR) data, Eustace et al. [14] took 50 as optimum segmentation scale-factor after three experiments by multiresolution segmentation. Combinations of attributes of the image objects (i.e., slope, texture, standard deviation of the slope, the length of longest edge of the object, length-to-width ratio, rectangular fit, asymmetry, and the relation to neighbours) were tested to determine the best thresholds and the attributes that most accurately classified gullies. Shruthi et al. [29] confirmed multiresolution segmentation scale factor, respectively, is 72 and 92 employing multiresolution segmentation mode and chessboard segmentation mode, based on orthorectified IKONOS data (5 bands: blue, green, red and NIR of 4 m resolution, and PAN data of 1 m resolution; P-B-G-R-NIR) and DSM data (Digital Surface Model generated by Stereoscopic GEOEYE-1 data) by ESP (Estimation of Scale Parameter) [34] and confirmed chessboard segmentation mode scale factor is 1 as a rule of thumb. Combinations of attributes of the image objects (i.e., SCA, areas, slope gradients, NDVI, GLCM, contrast filter mean values, mean Lee-Sigma, etc.) were tested to determine the best thresholds and the attributes to accurately classify segmented images as gully or nongully [29]. In complex hilly farming areas, due to man-made (such as sporadic fruit trees planted on north slope of Gully 1 in this study), natural, and other factors leading to chaos and complexity of gully system, spectral and texture information is quite complicated, and it is hard to interpret gully shoulder line effectively using the traditional method of visual interpretation. Theoretically, after careful analysis of the differences in the characteristics of the spectrum, geometry, texture, inheritance and adjacency between image objects, and through multiple segmentation-classification cycles, gullies can be effectively sorted out completely. In addition, due to different remote sensor performance, imaging environment and environment of study area, the classification features, and threshold selection still have a certain degree of subjectivity. However, compared to the traditional visual interpretation of vector gully mapping methodology, object-based analysis technology is more efficient with higher accuracy [29].

There are few effective methods to evaluate remote sensing classification accuracy of individual gully. Shruthi et al. [29] avoided individual classification accuracy test of gullies by taking visual interpretation and field survey as the inspection area (grid format) and adopted convex hull approach [35] to perform accuracy test for gully system classification result. This study, however, proposed a new method for testing boundary classification accuracy of small aged gullies based on adjacency approach. This method can be used in accuracy quantitative evaluation of classification boundary line which requires high classification accuracy, but it requires measured point data of gully edge line. The range difference calculated in the experiment primarily reflects the accuracy of segmentation and classification, but it may have slight errors brought in by the inconsistency of acquisition time of the DOM data and the GPS measuring data.

Sediment yield of gully erosion is affected by such factors as rainfall, hydrology, soil, time, and land use type. Comparatively correct recovery of the original terrain surface before gully occurs is the premise of the quantification of gully erosion volume. The recovery method of gully's original terrain surface directly plays a decisive role in the calculating precision of gully's water and soil loss. The gully erosion volume in certain time quantum is often accurately calculated through space overlay analysis in accordance to DEM data and topography data of fitted gully's original surface based on interpolation. Interpolation algorithm should be selected according to specific experimental zone terrain trend and the DEM data of other areas besides gullies. Eustace et al. [14] used adjacent pixel interpolation to fit gully original terrain surface and calculated the amount of gully soil erosion on the basis of the study area's DSM data. In this paper, considering that the sizes of the experimental gullies (Gully 1, Gully 2) are small and both sides of which are adjacent to agricultural land of the same topographic trend, therefore, planar fitting method is used in accordance with the elevation values of the gully's classification edge point to recover the gully's original surface and greatly decrease workload of field measuring elevation as well. Precision evaluation of this method has yet to be discussed in follow-up study. Besides, with the gully's remote sensing image and digital elevation model of different time, time series analysis of research gully morphological parameter could be conducted based on gully's original terrain surface recovery method proposed in this paper to accurately understand the occurrence, development process, rule, and mechanism of gully erosion.

## 5. Conclusions

Remote sensing technology has been widely used in gully erosion studies with the advantages of wide detection range, fast acquisition of information, short period, less restrictions by ground conditions, and rich electromagnetic wave information. The aerial photography and satellite of high spatial resolution have sprung up; for example, the spatial resolution of US commercial satellites, geoeye-1, worldview 1/2 series, and French pleiades satellite have reached 0.5 meter; spatial resolution of geoeye-2 and worldview-3 to be launched can reach 0.25 and 0.31 meter, respectively, which will provide more convenient and high-quality remote sensing data for the standardized production of DOM images. Characterized by geometric accuracy of map, image, and standard framing, DOM images offer abundant remote sensing images for a standardized and dynamic study of gully. Based on single pixel, traditional extracting technology of remote sensing information fails to fully unearth the information in DOM remote sensing images of high resolution. Based on very high spatial resolution, optical aerial photographs DOM image, this paper adopted object-oriented multiscale segmentation techniques and effectively extracted small watershed experimental gully edge line in Beiyanzikou ditch from the spectral information, geometry information (aspect ratio, area), and texture information (GLCM) of image objects. Then, it proposed a new accuracy evaluation method by computing the adjacent distance between GPS measured gully border points and the boundary of classification patterns. The results of accuracy evaluation show that the mean value of the extracted experimental gully error (deviation) is 0.3166 m and the standard deviation is 0.2116 m. Finally, based on the differential theory, it extracted classification boundary pixel elevation values of small gullies and performed linear regression to fit gullies original surface. Through calculating excavation volume between GPS measured DEM data and the fit original surface, erosion volume of Gully 1 and Gully 2 are obtained, which are 5074.1790 m^3^ and 1591.5784 m^3^, respectively. Fitting surface *R* ^2^ is 0.9461 and 0.8959 and RMS error is 0.5978 m and 0.6159 m, respectively, showing a high surface fitting accuracy. Results of this study provide a new method for a fast quantitative study of small gully erosion.

## Figures and Tables

**Table 1:** Basic information of the experiment data.

Data	Time	Spatial resolution (m)	Explanation
Aerial photo (DOM)	2008-5-3	0.25	Orthorectification image with visible wavelengths (red, green, and blue)
DEM	2012-3-4	0.25	RTK-GPS dynamic measurement, Figure 2; DEM Raster data, Figure 3.
Gully boundary	2012-3-4	—	Vector data, RTK-GPS dynamic measurement

**Table 2:** Aerial imagery RGB triband relevance statistics.

Correlation	Red band	Green band	Blue band	St. dev.
Red band	1			69.517062
Green band	0.936658	1		65.021132
Blue band	0.930912	0.887532	1	65.048784
